# The End of the X-Waiver: Not a Moment Too Soon!

**DOI:** 10.7759/cureus.15123

**Published:** 2021-05-19

**Authors:** Joseph Pergolizzi, Jo Ann K LeQuang, Frank Breve

**Affiliations:** 1 Cardiology, Native Cardio Inc., Naples, USA; 2 Pain Management, NEMA Research, Inc., Naples, USA; 3 Department of Pharmacy, Temple University, Philadelphia, USA

**Keywords:** buprenorphine maintenance therapy, buprenorphine, x-waiver, opioid use disorder, opioid rehabilitation

## Abstract

The X-waiver requires a physician to obtain specific training and federal certification to prescribe buprenorphine to treat opioid use disorder (OUD). Outgoing President Donald Trump issued an order that would have exempted many physicians from the X-waiver, but incoming President Biden stated they would repeal the Trump order despite campaign promises to better treat OUD. Opioid rehabilitation is a big business and a complex one, but there are not enough places in rehabilitation programs to meet demand, which is increasing year after year. In many cases, the X-waiver makes it more difficult and imposes more limitations on physicians to offer buprenorphine maintenance therapy than opioid prescribing. The Biden administration recently announced that it will not block the Trump orders, but rather will get rid of the X-waiver. The authors hope this is a permanent move away from the antiquated X-waiver. Further, this would provide greater access to accessible, affordable, and evidence-based OUD treatment to more patients, and may help break down some healthcare disparities in the treatment of drug disorders.

## Editorial

On January 14, 2021, outgoing President Donald Trump issued an order that would have exempted many physicians from the X-waiver, a 20-year-old requirement that physicians must obtain specific training and federally issued certification to prescribe buprenorphine to treat opioid use disorder (OUD). However, on January 28, 2021, news from the incoming Biden administration stated that Health and Human Services was on track to repeal the Trump order and continue the antiquated X-waiver, despite earlier promises that Biden would offer improvements in how we treat OUD. The current political confusion over the X-waiver generates frustration over our ability and commitment to use evidence-based tools to fight OUD. The X-waiver must be dropped, and there is no time to waste.

The opioid crisis is getting worse. From May 2019 to June 2020, over 83,000 people died in the United States from a drug overdose, the highest number in history and 21% over the prior year [1]. Most overdose deaths in this country involve an opioid. There is an urgent and unmet medical need to fight OUD with the best weapons in our arsenal. In 2018, over 164 million Americans over the age of 12 were prior-month substance abusers of some form (including alcohol, tobacco, and illicit drugs) and over 10 million misused opioids (prescription products as well as street drugs) in the preceding year [2]. While an estimated 21.2 million people in the United States over the age of 12 required some form of treatment for substance abuse in 2018, only 1.4% received any such care [2]. It is not known how many individuals with OUD want rehabilitation services, nor is it clear how many would seek rehabilitation if non-stigmatized care were available, accessible, and affordable.

Meanwhile, opioid rehabilitation has become both a big business and a black box. Drug and alcohol rehabilitation in the United States is a $42 billion industry plagued by accusations of ineffectiveness, overpricing, insurance fraud, and lack of transparency [3]. There are about 14,000 rehabilitation centers in the United States offering residential services, but insurance restrictions, prohibitively high pricing even with insurance, location, and space limitations mean that many who might want care have no access. Even some who can afford these high-end solutions are wait-listed. Despite the demand for rehabilitation, these centers are surprisingly opaque about their effectiveness and some rely on mutual support efforts such as 12-step programs, which are often available free in the community setting.

In 2016, the Centers for Disease Control and Prevention issued guidance to family care physicians about opioid prescribing and recommended that clinicians should refer or oversee patients with OUD for medication-assisted opioid rehabilitation therapy with buprenorphine or methadone, further adding that family practice physicians prescribing opioids should consider obtaining an X-waiver to prescribe buprenorphine maintenance. Methadone maintenance for opioid rehabilitation has been available for 50 years, and while it still remains strangely controversial in some circles, it is an effective and evidence-based treatment. Treating OUD with a combination product of buprenorphine plus naloxone (Suboxone®) is a newer, safe, effective, and evidence-based therapy that can be administered to outpatients, but this use is limited by the X-waiver and patient treatment counts it imposes. Current standards allow a physician with an X-waiver to treat 30 patients in the first year, up to 100 patients in following years providing federal approval is obtained, and even board-certified addiction specialists are not allowed to treat more than 275 patients at a time. No such restrictions are imposed on opioid prescribing. Because buprenorphine maintenance therapy is often a long-term or lifelong treatment for OUD, physicians interested in treating OUD patients may quickly reach maximum limits and be unable to add new patients.

The X-waiver seems to regard opioid rehabilitation as more dangerous than opioid prescribing. After all, appropriately licensed prescribers can prescribe opioids to their patients but without an X-waiver would be disallowed from offering buprenorphine maintenance. Buprenorphine maintenance offers several advantages over the older methadone maintenance. Unique among opioids, buprenorphine has a ceiling on respiratory depression [4] and is a Schedule III controlled substance (unlike morphine, methadone, or oxycodone, which are Schedule II) and not often taken by recreational users for its psychoactive purposes. Indeed, buprenorphine diversion, which does occur, is mainly carried out by individuals who are trying to manage opioid withdrawal and rehabilitation on their own rather than seeking the drug’s psychoactive effects. [5] Those who do use buprenorphine for recreational purposes report that it is not their drug of choice but may be taken in the event no other opioid was available [5].

The DEA proposal to lift the ban on methadone mobile vans is indicative of the need to increase access to OUD treatment for patients in rural areas. This same rationale should be applied to the case of buprenorphine by allowing clinicians not to be burned with prescribing restrictions. This provides OUD patients full and unfettered access to all evidence-based treatment modalities for the best possible outcome.

It is long past time to abandon the X-waiver once and for all and to make evidence-based opioid rehabilitation available to all who want it, and to open up clinical practices for safe, affordable, and community-based care for those struggling to overcome OUD. Seeing the current administration trying to go backward by not repealing the X-waiver was disheartening, and while this lifting of the X-waiver inspires some optimism, we need confidence that the X-waiver will no longer be a hurdle to offering treatment to those trying to overcome OUD. When OUD treatment is readily available, affordable, welcoming, non-stigmatized, non-judgmental, and evidence-based, it is likely that more patients will seek treatment and more patients will recover. Most of all, getting rid of the X-waiver will deal a blow to some of the healthcare disparities that plague this nation by expanding evidence-based OUD treatment to include poor patients, patients without access to opioid rehabilitation centers, patients of color, immigrants, rural patients, and the growing number of women with OUD who have children or other family commitments that preclude residential care, even if they could afford it. Removing the X-waiver helps to de-stigmatize OUD by removing some of the barriers to equitable, accessible, affordable, evidence-based treatment. OUD is a medical problem and requires a medical solution, not a bureaucratic one. We hope that the Biden administration will get rid of the X-waiver for good.
